# Oral cancer in young adults: should we approach these patients differently?

**DOI:** 10.3389/fonc.2024.1297752

**Published:** 2024-04-05

**Authors:** Mateusz Szewczyk, Jakub Pazdrowski, Paweł Golusiński, Barbara Więckowska, Wojciech Golusiński

**Affiliations:** ^1^ Department of Head and Neck Surgery, Poznań University of Medical Sciences, The Greater Poland Cancer Center, Poznań, Poland; ^2^ Department of Otolaryngology and Maxillofacial Surgery, University of Zielona Góra, Zielona Góra, Poland; ^3^ Department of Computer Science and Statistics, Poznan University of Medical Sciences, Poznan, Poland

**Keywords:** oral, oral cancer, young, young adults, head neck, head neck cancer

## Abstract

**Objective:**

The influence of age on treatment outcomes in oral cancer is unclear. We aimed to determine the prevalence of oral cancer in adults under age 45 and to compare treatment outcomes by age.

**Methods:**

Retrospective study of 284 patients treated for oral cancer from 2010 to 2021. The primary analysis involved the full cohort stratified by age (< vs. ≥ 45y). The second analysis included all patients under age 45 (n=44) matched 1:1 by sex and stage to older patients (age 55-70).

**Results:**

In the primary analysis, the only significant difference was more comorbidities in the older group (p<0.001). In the matched-pair analysis, older patients were more likely to be smokers (75% vs. 54%; p=0.045) and had more comorbidities (p=0.007). The mean PLR and NLR values were significantly higher in the younger group.

**Conclusions:**

No significant differences were observed between age groups in disease stage or outcomes, suggesting that other variables are more important.

## Introduction

Oral cancer generally affects men in the 6^th^ and 7^th^ decades of life, predominantly smokers and heavy drinkers (1). However, in recent decades, the incidence of oral cancer has been rising among younger patients (age < 45), increasing from 3%-5% in the 1970s and 1980s to approximately 10% at present (2–5). Crucially, the usual risk factors—tobacco and alcohol use—are, in many cases, not present in these younger patients (6–9). Although the reasons underlying the growing incidence of oral cancer in younger patients remain unknown, recent data suggest that it might be related to an impaired immune system (10–12).

Although some studies have found that young adults have worse survival outcomes, other studies have reported contradictory findings (i.e., poorer survival among older patients), which suggests that age may not be a reliable prognostic indicator (13–17). Given these inconsistent data, the influence of age on treatment outcomes in patients with oral cancer remains unclear.

In this context, the main aim of the present study was to determine the prevalence of oral cancer in a cohort of younger adults treated at our hospital. A second aim was to compare treatment outcomes between younger and older adults (< or ≥ 45). Finally, we sought to identify the risk factors present in young adults who developed oral cancer.

## Materials and methods

This was a retrospective study of 284 patients diagnosed and treated for oral squamous cell carcinoma from 2010 to 2021 at our institution. The only inclusion criterion was a diagnosis of primary oral cancer during the study period (2010-2021). Exclusion criteria were: lip cancer; recurrent disease; previous treatment for head and neck cancer; incomplete medical history; and/or < 12 months of follow up (except for patients who died).

All patients underwent primary surgical resection with ≥ 1 cm macroscopic tumour-free margins (both lateral and deep). In patients without clinical nodal involvement (N0), elective neck dissection was performed (nodal levels I – III, unilateral or bilateral in midline tumours). In patients with node-positive neck disease, therapeutic neck dissection (levels I - IV/V, as appropriate) was performed. Following surgery, all patients were evaluated by a multidisciplinary team to determine patient eligibility for adjuvant radiotherapy and/or chemotherapy.

The standard radiotherapy protocol was 60–66 Gy (2.0 Gy/fraction) administered daily from Monday–Friday for 6 to 7 weeks. Eligibility requirements for adjuvant radiotherapy were as follows: stage pT3/4 disease; close surgical margins (1-5 mm); positive lymph nodes; and evidence of perineural or vascular invasion. The indication for chemotherapy included positive surgical margins and/or extranodal involvement. The chemotherapy regimen consisted of concurrent, single-agent cisplatin (100 mg/m^2^) administered every 3 weeks.

The following clinical parameters were registered: age at diagnosis; sex; smoking habit (pack years); alcohol use; comorbidities (Charlson Comorbidity Index); disease stage; T status; N status; number of positive metastatic lymph nodes; perineural invasion (PNI); lymphovascular invasion (LVI); extranodal extension (ENE); and final margin status. The type of recurrence (local, regional, and/or distant) and/or type of second primary tumour were also assessed. Disease-free survival (DFS) and overall survival (OS) rates were calculated. The primary analysis included the entire group stratified by age (< or ≥ 45 years). These two groups were then compared to assess for differences in clinical parameters.

### Matched pair analysis

All patients under age 45 (n=44) were matched 1:1 with older patients (age 55 to 70 years) by local/regional disease stage. Patients aged 46–55 years were excluded from this analysis because the incidence of oral cancer in this group is closer to that of the younger patients. For matching purposes, the local disease stage was dichotomized into stage T1-2 vs. T3-4, and regional stages into N0 vs. N+. Next, the groups were compared according to the following clinical and demographic variables: smoking and alcohol history; comorbidities; tumour grade; PNI; LVI; ENE; adjuvant treatment; final surgical margin status; recurrence (yes/no); type of recurrence; neutrophil-to-lymphocyte ratio (NLR); and platelet-to-lymphocyte ratio (PLR). Differences were calculated using Pearson’s chi-square test and Fisher’s exact test. Statistical significance was set at p < 0.05. Survival was calculated using Cox-proportional hazard models. In addition, we adjusted the results for identified confounders. For this purpose, we used multivariate logistic regression models and multivariate Cox proportional hazards models.

Considering retrospective study design and anonymized data reporting, ethics approval was not considered necessary by the local committee. Every patient that has been admitted to our Department has signed a consent for collecting information.

## Results

Most of the patients were males (188/284; 66%). The mean (standard deviation [SD]) patient age was 58 (11.9) years (range, 23–97). Most of the patients (n=240; 84.5%) were ≥ 45 years of age; the remaining 44 patients were < 45 years. The two most common tumour locations were the tongue (n=130, 45%) and floor of mouth (n=98, 34%), accounting for 89% of all the cancer diagnoses.

At baseline, the statistically significant differences between the groups were in the number of comorbidities (more prevalent in the older group (p<0.001)) and smoking status (more pack years in older group (p<0.0001)). A trend toward significance was observed in N status (p=0.08), with a higher percentage of older patients presenting with N0 disease (59% vs. 50%) (**Table 1**).

**Table 1:** Demographic and clinical variables by age group at baseline.

Variable	Total 284	≥ age 45 (n=240)	< age 45 (n=44)	p value	p value adjusted
n (%)					
**Male**	188 (66%)	158 (66%)	30 (68%)	0.7621	0.642
**Active smoker (%); Mean pack years**	171 (60,21); 35,9 ± 9.4	147 (61,25); 38,2 ± 7.4	24 (54,55); 21,6 ± 7.9	**<0.0001**	0.3545
**Heavy drinker (3/4 drinks per day for females/males)**	42 (15%)	36 (15%)	6 (14%)	0.8148	NA
**Comorbidities, Charlson Comorbidity Index (mean)**	4,45 ± 1.63	4,82 ± 1.46	2,41 ± 0.72	**<0.0001**	**<0.0001**
**T stage**				0.8209	0.4493
1	87 (31%)	75 (31%)	12 (27%)		
2	136 (48%)	111 (46%)	25 (57%)		
3	44 (15%)	38 (16%)	6 (14%)		
4	17 (6%)	16 (6%)	1 (2%)		
**N stage**				0.0815	0.0986
N0	164 (58%)	142 (59%)	22 (50%)		
N+	120 (42%)	98 (41%)	22 (50%)		
%					
**Disease stage (I, II, III, IV)**	(24/24/23/29)	(25/25/23/27)	(23/20/18/39)	0.2748	0.1451
**Tumour grade (1/2/3)**	(21/63/16)	(21/63/16)	(18/61/21)	0.4208	0.3355

p value adjusted for smoking status and Comorbidities, Charlson Comorbidity Index.

NA -not calculated due to insufficient subgroups sizes or high correlation between variables in the model.

Bold value stands for p value that is statistically significant.

In the matched pair analysis, a higher proportion of older patients were smokers (23,92 pack years vs 12,36, p<0.0001), with more comorbidities in the older group (Charlson Comorbidity Index 5 vs 2, p<0.0001). The mean (SD) PLR and NLR values were significantly higher in younger versus older adults (157.72 [64.8] vs 118.24 [41.29], p<0.001; and 3.19 [1.52] vs 2.70 [1.88], p=0.018, respectively). No significant between-group differences were observed for any of the following variables: tumour grade; PNI; ENE; LVI; final surgical margin status; use of adjuvant RT/CRT; recurrence (**Table 2**).

**Table 2:** Results of matched pair analysis.

Variable	Age 55 to 70 years (n=44)	Age < 45 years (n=44)	p value	p value adjusted
	n (%)			
**Active smoker (%);** **Mean pack years**	33 (75); 41,03 ± 4.36	24 (54); 21,67 ± 7.94	**<0.0001**	0.6151
**Heavy drinker (3/4 drinks per day for females/males)**	2 (5%)	5 (11%)	0.4336	NA
**Comorbidities, Charston Comorbidity Index, mean**	4,45 ± 0,97	2,41 ± 0,72	**<0.0001**	**<0.0001**
**NLR**	2.7 ± 1.88	3.19 ± 1.52	**0.018**	0.3952
**PLR**	118.24 ± 41.29	157.72 ± 64.8	**<0.001**	**0.0289**
**Tumour grade, % (1/2/3)**	(29/52/19)	(18/61/21)	0.326	0.1633
**PNI +**	5 (11%)	12 (27%)	0.0587	0.3909
**LVI +**	4 (9%)	3 (7%)	1	0.4058
**ENE+**	9 (20%)	11 (25%)	0.6519	0.7299
**Positive margins**	10 (23%)	9 (20%)	0.7956	0.4344
**Any recurrence**	18 (41%)	19 (43%)	0.829	0.8061
**If recurrent, salvage possible? Yes**	2 (11%)	6 (31%)	0.1212	NA
**Isolated distant metastases**	4 (22%)	2 (10%)	0.3828	NA

NLR, neutrophil-to-lymphocyte ratio; PLR, platelet-to-lymphocyte ratio; PNI, perineural invasion; LVI, lymphovascular invasion; ENE, extranodal extension.

p value adjusted for smoking status and Comorbidities, Charlson Comorbidity Index.

NA -not calculated due to insufficient subgroups sizes or high correlation between variables in the model.

Bold value stands for p value that is statistically significant.

**Figure 1:**
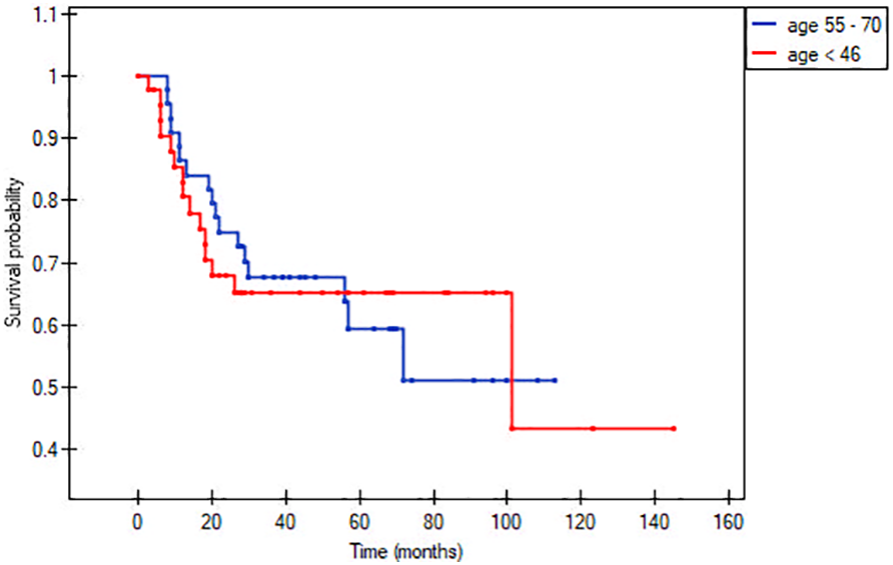
Overall survival according to age (< 45 vs. 55-70 years).

**Figure 2:**
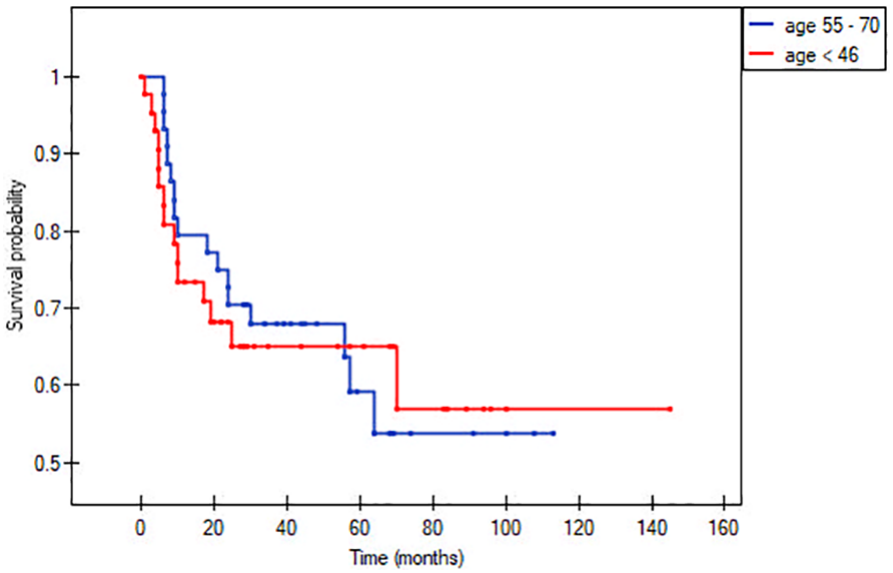
Disease-free survival according to age (< 45 vs. 55-70 years).

**Figure 3:**
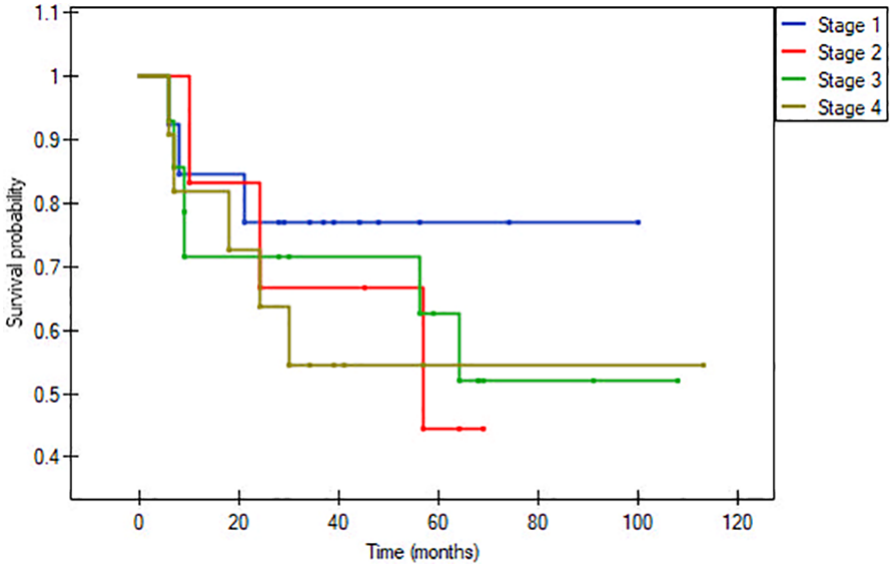
Disease-free survival within older patients group, p= 0.7811.

**Figure 4:**
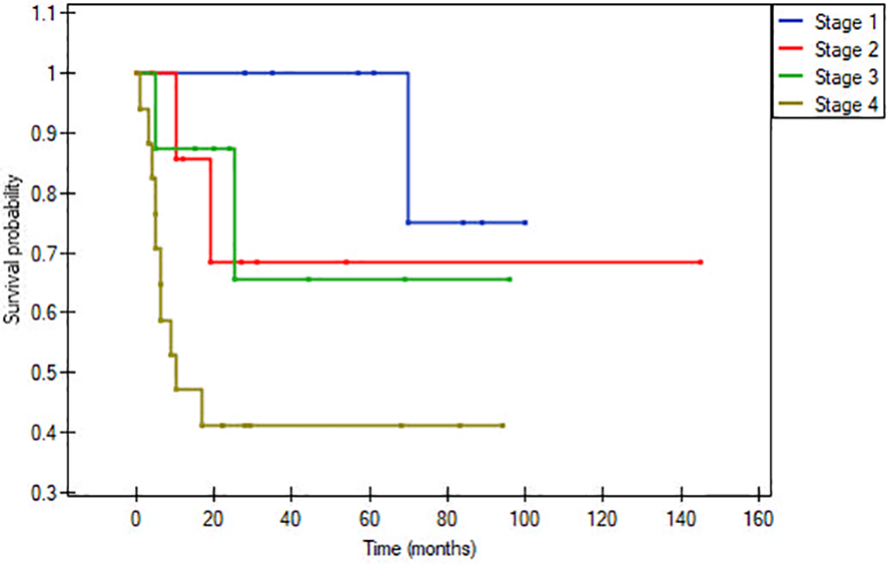
Disease-free survival within young adults, p= 0.02864.

In the matched pair analysis, no between-group differences were observed in survival outcomes (OS and DFS) (**Figures 1**, **2**). Differences were noted in DFS within subgroups (young adults and adults) stratified by stage (**Figures 3**, **4**). No survival differences (data not shown) were observed between subgroups when stratified by T stage, N stage, disease stage, number of positive metastatic lymph nodes, PNI, LVI, ENE and positive surgical margins. ROC curves could not be calculated according to PLR and NLR values due to the limited number of patients in the subgroups.

## Discussion

This study was conducted to determine whether there are significant clinical and/or demographic differences between younger and older patients diagnosed with oral cancer. In the full cohort (n=284, primary analysis), the only significant differences between these groups in our study was that older patients had more comorbidities (based on Charlson Comorbidity Index) and had more pack years of smoking (**Table 1**). On the matched pair analysis, the percentage of active smokers among the older group was significantly higher (75% vs. 54%; 41,03 vs 21,67 pack years). Survival outcomes at 5-years were similar in the two groups: OS: 60% vs. 65% and DFS: 58% vs. 66%, respectively (**Figures 1**, **2**). NLR and PLR values were both significantly greater in the younger patients.

In recent decades, the patient profile in oral cancer has changed substantially, with an increase in the incidence of oral cancer in patients under age 45, many of whom neither smoke nor drink heavily (2, 5, 7, 18, 19). The available data shows heterogenous treatment outcomes in these younger patients, but the reason for these mixed results is not clear. For example, Friedlander et al. found that, compared to older patients, recurrences in younger patients were more likely to be local or regional, although this difference was not statistically significantly (13). More recently, Kaminagakura and colleagues found a higher recurrence rate in younger patients (20). By contrast, both Oliver et al. and Oh et al. found better outcomes among younger patients (14, 15).

In our sample, the only significant clinical differences between the older and younger groups were a greater number of comorbidities in the older group and pack years of smoking. However, this finding was not unexpected given that the prevalence of chronic disease increases with age. In a recent study by Jones et al. the authors compared younger and older patients, non-smokers non-drinkers and noticed no significant patient characteristic differences between subgroups apart from comorbidities (similar to our study) but older patients were more often in higher disease stage (21). Although some studies (4, 22) have found that oral cancer is more common in younger women than in men, our data show the opposite, with men accounting for 68% of patients in that subgroup (patients under age 45). Other studies, such as the one performed by Oh et al., have found differences in nodal status between older and younger patients, with young adults more likely to be node positive (14). Although we also observed a small difference in nodal status between groups (**Table 1**), this difference was not statistically significant.

The carcinogenic effects of tobacco use are well-known and smokers have a 10-fold greater risk of developing head and neck cancer than non-smokers (23). However, exposure to the carcinogen must be sufficiently prolonged to lead to carcinogenesis, which would explain why smoking is not necessarily associated with oral cancer in younger smokers (24). Surprisingly, Lee at al. found that younger patients who smoked tobacco and drank alcohol had better survival outcomes than younger patients who did not smoke or drink (25). Although this finding appears contradictory, the authors hypothesized that younger patients with less smoking/alcohol exposure who develop oral cancer have a specific tumour biology. We did observe age-related differences in tobacco use in our full cohort in terms of pack years; however, on the matched pair analysis, 75% of patients aged 55-70 were smokers versus only 54% of patients under age 45 (23,92 vs 12,36 pack years, p<0.0001). In this regard, other studies have reported heterogenous data, with Kaminagakura et al. (20) finding no differences in tobacco use between age groups while Farquhar et al. found that younger patients were less likely to use tobacco (22). In a recent review by Batistella et al. on over 15 000 pts, younger oral cancer patients tend to smoke and drink less than older counterparts (26). An interesting study regarding tobacco use and oral cancer in young adults was published by Subramaniam et al. The authors examined the effect of tobacco use on survival stratified by age and concluded that tobacco use was an independent predictor for recurrence only in younger patients subgroup (27).

We did not observe any between-group differences in our cohort in initial disease stage, a finding that is consistent with the reports by van Monsjou et al. and Oliver et al. (15, 28). By contrast, Farquhar et al., Oh et al. and Jones et al. (14, 21, 22) found that younger patients were more likely to present with early stage disease.

A secondary aim of our study was to determine whether age influences treatment outcomes. To evaluate this, we performed a matched pair analysis by T stage (stages 1 + 2 and 3 + 4) and N stage (N0 and N+ stage). No significant survival differences were observed between any of these subgroups. Overall survival rates at 5-years were similar in the two groups (60% in older patients vs. 65% in younger patients). Similarly, no survival differences were observed when the groups were stratified by clinicopathological risk factors (**Table 2**).

In the literature, contradictory data have been reported with regards to the role of age on survival outcomes. In a most recent study Jones et al. have compared only non-smokers non-drinkers young and old patients and have discovered that although young patients had worse distant metastasis free survival (62,8% vs 88,1% in older pts group) it had not affected the disease specific survival which was similar between subgroups (21). In another large review by Kaminagakura et al. the authors analysed over 1500 pts (12 studies under analysis) and concluded that younger patients had worse disease free survival (pooled hazard ratio of 0.73) (29). Garavello et al. found that both recurrence rates and survival outcomes were worse in young patients (30). In that study, there were no significant clinical differences between the groups, leading the authors to hypothesize that oral cancer in young adults may have a distinct biological behaviour. Friedlander et al. reported higher locoregional failure rates in younger patients but no between-group differences in OS (13). Kaminagakura and colleagues in separate study found higher recurrence rates in younger patients, with a shorter time to recurrence compared to older patients, but no impact on OS (20). Farquhar et al. (22) obtained similar results, with younger patients having a three-fold greater risk of recurrence, but with no impact on OS. Younger patients in that study were less likely to be smokers, which supports the notion that tobacco use does not appear to play a significant role in treatment outcomes. The study by Farquhar and colleagues is the only study in which pathological risk factors (e.g., PNI and LVI) were more prevalent in younger patients; nevertheless, the authors concluded that oral cancer in young adults has a distinct biology and phenotype. In a more recent study, Oliver et al. found that younger patients had significantly better survival outcomes despite presenting higher rates of nodal metastases and LVI (15). Finally, in a large multicentre study of patients (n=3818) with oral cancer, age was not a predictor of disease-specific mortality; rather, the main variable influencing treatment outcomes was the number of adverse features (14).

In recent years, the incidence of oral cancer has increased, especially among non-smokers; however, the reasons underlying this change remain poorly understood. In a recent review by Tran et al. the authors examined oral cancer in non-smokers non-drinkers young patients. When discussing aetiology of the disease the authors mentioned among others genomics, microbiome (increase in Fusobacterium, Mogibacterium and Tannarella) and viruses (EBV and HPV) but without strong evidence-based data (31). Kim et al. in their recent study also examined the genetic alterations in young adults diagnosed with oral cancer. Their study demonstrated that young patients with advanced stage of disease had more frequent TERTp mutations in comparison to older counterparts and it did negatively impact patients prognosis. The authors suggested that this could be a new biomarker for patients requiring more aggressive treatment (32). Most studies suggest that the biological behaviour of oral cancer is different in younger patients, a hypothesis that is supported by the higher recurrence rates in these patients, despite the lower smoking rates in this group and despite the fact that both older and younger patients share similar pathological features. One interesting hypothesis to explain this phenomenon could be immune system impairment in some younger patients. Valero et al. (33) divided a cohort of oral cancer patients into four subgroups according to age and smoking status, as follows: young adult smokers, young adult non-smokers, adult smokers, and adult non-smokers. After adjusting for tumour-specific and host factors, they found that young non-smokers had a higher risk of death, recurrence, and distant metastases. The authors suggested that this finding could be due to immune dysregulation, as the young non-smokers had a higher NLR than controls. Data from several other studies also support this hypothesis. For example, Wang et al. carried out a large metanalysis that showed that a high NLR—an indicator of systemic inflammation—negatively impacted both disease-specific survival and OS in patients with oral cancer (11). In another large study of patients with oral cancer (n=1369), Valero et al. obtained similar findings: patients with higher NLR treated for head and neck cancer had worse local, regional, and distant recurrence-free survival (10). In our study, NLR values were significantly higher in younger patients, which provides further support for this hypothesis, suggesting that inflammation negatively affects immune system function, which in turn increase the likely of developing oral cancer and also negatively impacts survival rates in these patients. Even though age and smoking are both associated with higher NLR values, this ratio was higher in our younger patients, who smoked less than their older counterparts (12, 34). Similarly, the PLR was significantly higher in the younger patients in our series. However, due to small number of patients in these subgroups, we were not able to draw ROC curves to assess the impact of these ratios on survival. In the metanalysis carried out by Zhang and colleagues to examine the negative effects of PLR on survival, a higher PLR was associated with significantly worse OS (OR=2.06) and disease-specific survival rates (OR=2.12) (35). Our findings provide additional data pointing to the involvement of immune dysfunction in the development of oral cancer in young adults. Nonetheless, large, multicentre studies are needed to verify these findings.

### Strengths and limitations

The main limitation of this study is the retrospective design. In addition, the limited number of patients in the matched pair analysis, particularly in the various subgroups, was insufficient to obtain statistically significant data. Nonetheless, this study provides additional data from a tertiary cancer care centre to better characterize the clinical features of younger patients and differences in treatment outcomes.

## Conclusion

In contrast to some previous reports, the present study shows that older and younger patients with oral cancer are similar in terms of initial disease stage and treatment outcomes. Although older patients tend to smoke more, this does not appear to significantly impact treatment outcomes. The reasons underlying the growing incidence of oral cancer in younger patients remain poorly understood, although data from several studies, including ours, suggest that it might be related to an impaired immune system. Nevertheless, more research is needed to better understand this phenomenon.

## Data availability statement

The original contributions presented in the study are included in the article/supplementary material. Further inquiries can be directed to the corresponding author.

## Ethics statement

Ethical approval was not required for the study involving humans in accordance with the local legislation and institutional requirements. Written informed consent to participate in this study was not required from the participants or the participants’ legal guardians/next of kin in accordance with the national legislation and the institutional requirements.

## Author contributions

MS: Formal Analysis, Methodology, Writing – original draft. JP: Methodology, Supervision, Writing – review & editing. PG: Investigation, Writing – original draft. BW: Formal Analysis, Methodology, Resources, Writing – review & editing. WG: Supervision, Writing – review & editing.
